# Diagnostic separation of conventional ⩾50% carotid stenosis and near-occlusion with phase-contrast MRI

**DOI:** 10.1177/23969873231215634

**Published:** 2023-11-30

**Authors:** Madelene Holmgren, Alexander Henze, Anders Wåhlin, Anders Eklund, Allan J Fox, Elias Johansson

**Affiliations:** 1 Department of Clinical Sciences, Neurosciences, Umeå University, Umeå, Sweden; 2 Department of Radiation Sciences, Biomedical Engineering, Umeå University, Umeå, Sweden; 3 Umeå Center for Functional Brain Imaging, Umeå University, Umeå, Sweden; 4 Department of Applied Physics and Electronics, Umeå University, Umeå, Sweden; 5 Sunnybrook Health Science Center, University of Toronto, Toronto, ON, Canada; 6 Wallenberg Center for Molecular Medicine, Umeå University, Umeå, Sweden; 7 Neuroscience and Physiology, Sahlgrenska Academy, Gothenburg, Sweden

**Keywords:** Carotid stenosis, near-occlusion, phase-contrast MRI, CT angiography

## Abstract

**Introduction:**

The aim of this study was to assess sensitivity, specificity and interrater reliability of phase-contrast MRI (PC-MRI) for diagnosing carotid near-occlusion.

**Patients and methods:**

Prospective cross-sectional study conducted between 2018 and 2021. We included participants with suspected 50%–100% carotid stenosis on at least one side, all were examined with CT angiography (CTA) and PC-MRI and both ICAs were analyzed. Degree of stenosis on CTA was the reference test. PC-MRI-based blood flow rates in extracranial ICA and intracranial cerebral arteries were assessed. ICA-cerebral blood flow (CBF) ratio was defined as ICA divided by sum of both ICAs and Basilar artery.

**Results:**

We included 136 participants. The ICAs were 102 < 50% stenosis, 88 conventional ⩾50% stenosis (31 with ⩾70%), 49 near-occlusion, 12 occlusions, 20 unclear cause of small distal ICA on CTA and one excluded. For separation of near-occlusion and conventional stenoses, ICA flow rate and ICA-CBF ratio had the highest area under the curve (AUC; 0.98–0.99) for near-occlusion. ICA-CBF ratio ⩽0.225 was 90% (45/49) sensitive and 99% (188/190) specific for near-occlusion. Inter-rater reliability for this threshold was excellent (kappa 0.98). Specificity was 94% (29/31) for cases with ⩾70% stenosis. PC-MRI had modest performance for separating <50% and conventional ⩾50% stenosis (highest AUC 0.74), and eight (16%) of near-occlusions were not distinguishable from occlusion (no visible flow).

**Conclusion:**

ICA-CBF ratio ⩽0.225 on PC-MRI is an accurate and reliable method to separate conventional ⩾50% stenosis and near-occlusion that is feasible for routine use. PC-MRI should be considered further as a potential standard method for near-occlusion detection, to be used side-by-side with established modalities as PC-MRI cannot separate other degrees of stenosis.

## Graphical abstract

**Graphical abstract:**
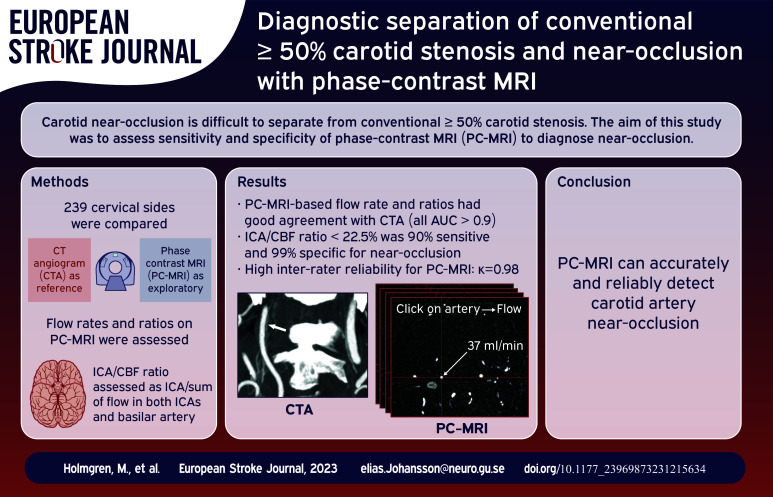

## Introduction

**Figure 1.**
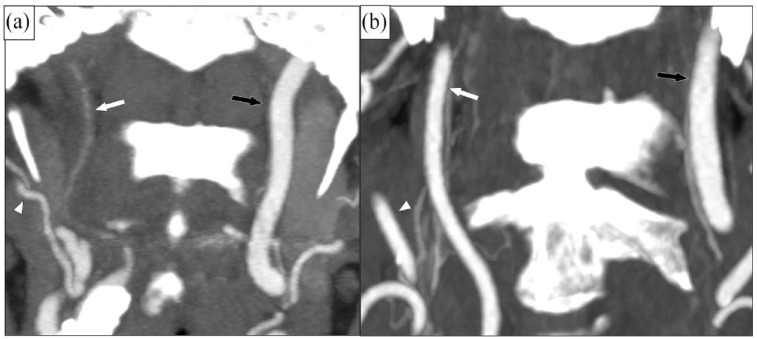
Two cases of right-sided near-occlusion. Coronal CTA. (a) Near-occlusion with full collapse. Beyond a severe stenosis (not in plane), the distal ICA (white arrow) has a threadlike appearance (approximately 0.7 mm diameter), clearly smaller than contralateral distal ICA (black arrow, 4.5 mm diameter) and ipsilateral ECA (white arrowhead, 3.0 mm diameter). ICA ratio 0.16, ECA-ratio 0.23. On PCI-MRI: Ipsilateral ICA flow not detectable, contralateral ICA 330 ml/min, ICA-CBF ratio 0. (b) Near-occlusion without full collapse. Beyond a severe stenosis (not in plane), the distal ICA (white arrow, 2.8 mm diameter) is normal-appearing but smaller than contralateral distal ICA (black arrow, 4.5 mm diameter) and similar to ipsilateral ECA (white arrowhead, 2.1 mm diameter). ICA ratio 0.62, ECA-ratio 1.33. On PC-MRI: Ipsilateral ICA flow 37 ml/min, contralateral ICA 330 ml/min, ICA-CBF ratio 0.076.

Carotid near-occlusion is a severe carotid stenosis that causes the distal internal carotid artery (ICA) diameter to reduce (“collapse”).^1^ The extent of distal ICA collapse can vary from severe, with a threadlike distal lumen (full collapse, previously called “string sign,” Figure 1(a)), to more modest, with a normal-appearing distal lumen (without full collapse, Figure 1(b)).^1^ In contrast, we define conventional carotid stenoses as those that do not cause distal ICA collapse. Latest European Society of Vascular Surgery guidelines recommend carotid revascularization for symptomatic conventional ⩾50% carotid stenosis, but that revascularization for symptomatic near-occlusions may only be considered for patients with repeated symptoms after multidisciplinary review.^2^ Latest European Stroke Organization Guidelines has no management recommendation for symptomatic near-occlusion given the diagnostic issues.^3^

Separating various degrees of conventional stenoses is feasible in routine practice as this is based on diameter measurements (angiographic modalities such as computed tomography angiography, CTA) and velocity thresholds (ultrasound).^2^ However, near-occlusion diagnostics in large trials and prognostic studies have been based on systematic feature interpretation.^1,4–7^ While collaborating experts can archive good reliability (kappa 0.80),^6^ this approach is seldom used in routine practice^8^ and has limited feasibility in routine practice, as there are no clear thresholds or rules. Suggested angiographic thresholds have been suggested by one recent guideline^2^ but is approximately 80%–90% sensitivity and specificity when applied in a manner resembling routine practice.^4,9^ Low-flow findings on ultrasound has very poor sensitivity as most near-occlusions have high stenosis velocity.^10,11^ Promisingly, a recent study suggested that thresholds of ICA flow ⩽110 ml/min and ICA flow rate ratio (ipsilateral/contralateral) <0.35 measured with phase-contrast MRI (PC-MRI) were both 100% accurate for diagnosing near-occlusion.^12^ However, this was a proof-of-concept study with a limited sample size (*n* = 29), requiring further validation.^12^

In this study, the aim was to investigate the efficacy of PC-MRI based diagnosis of carotid near-occlusion by establishing the sensitivity, specificity and interrater reliability of the method in an adequately powered sample.

## Methods

### Study cohort

The study setting was the stroke unit and Department of Radiology at the University Hospital of Northern Sweden, a tertiary center serving 11 referring hospitals. We prospectively included patients with an initial diagnosis of ⩾50% carotid stenosis or occlusion in at least one ICA, albeit a few (*n* = 3) had <50% stenosis bilaterally on the subsequent study CTA assessment. Most participants were assessed for possible symptomatic ⩾50% carotid stenosis, but persons with known asymptomatic ⩾50% carotid stenosis were also summoned for the study. Exclusion criteria were inability to undergo CTA (kidney failure and contrast allergy), MRI (significant body metal, non-compatible pacemaker, unable to move from wheelchair to table, and claustrophobia), and inability to provide informed consent. The study was conducted between February 2018 and November 2021, with a 6-month break in 2020 due to the Covid-19 pandemic. While a consecutive sample was sought, this was occasionally hindered by logistical challenges, such as short time to surgery and staff shortages. All participants underwent CTA and PC-MRI, most underwent carotid ultrasound. Preoperative recurrent stroke was assessed as in a previous study.^6^ The study was approved by the regional ethics board in Umeå, Sweden, in accordance with the declaration of Helsinki.

### CTA

All CTAs were assessed by observer 1 (EJ, 10 years’ experience). All with suspicion of near-occlusion and a similar number of controls before the Covid19-break by observer 2 (AJF, >40 years’ experience), and all after the break by observer 3 (AH, 5 years’ experience). Evaluators were blinded to each other, MRI findings, and postoperative CTA. Disagreements were resolved by consensus discussions.

Near-occlusion was the pre-specified primary outcome, diagnosed using systematic feature interpretation when the distal ICA was reduced in diameter, and the stenosis was the most reasonable cause.^6–8^ See data supplement for description. A two-sided conservative approach was used, both near-occlusion and conventional stenosis was diagnosed when features was sufficiently clear; ICAs were categorized as *unclear diagnosis* when the distal ICA was small of unclear cause (near-occlusion or anatomical variation), or it was uncertain if the distal ICA was small (varying diameter or severe stenoses with contralateral near-occlusion or occlusion). Among near-occlusions, full collapse was defined as ⩽2.0 mm distal ICA diameter and/or ⩽0.42 ICA ratio.^2,13^ Conventional stenoses were graded by comparing stenosis diameter with distal ICA diameter (NASCET method).^1,2^ Occlusion was defined as no visible flow beyond the stenosis, often using delayed CTA or conventional angiography to detect minute flow. *Conventional stenosis*, the main control group, was the combination of <50% and conventional *⩾*50% stenosis. To clarify our nomenclature: Near-occlusion is a degree of stenosis above the percent degrees: Thus, “⩾50% stenosis” or “⩾70% stenosis” includes near-occlusion, but “conventional ⩾50% stenosis” or “<50% stenosis” does not.

We also used postoperative CTA as a supplementary reference method, see data supplement.

### Phase-contrast MRI

Blood flow rates in arterial segments were assessed from four-dimensional PC-MRI.^14–16^ As only time-averaged data were analyzed, in practical terms we used three-dimensional PC-MRI. PC-MRI is used to quantify flow, a different approach to the luminal assessments of time-of-flight magnetic resonance angiography (MRA) and contrast-enhanced MRA. See data supplement for scanning and work-flow details, summarized in the central illustration. All flow rates were assessed by Observer 4 (MH, engineer, limited neuroradiological experience), with Observer 1 assessing flow rates in ICA and BA.

Mean blood flow rates were assessed in 15 arterial segments: bilaterally in extracranial ICA, middle cerebral arteries at M1 level, anterior cerebral arteries at A1 and A2 level, posterior cerebral arteries at P1 and P2 level, and posterior communicating arteries (Pcom), and in the basilar artery (BA) at the midpoint between vertebral artery confluence and the posterior cerebral artery bifurcation. Lateral flow rate ratios for ICA, M1, A1, P1, and P2 were calculated as the ratio of the ipsilateral- and the contralateral flow rate. Total cerebral blood flow (CBF) was defined as the sum of both ICAs and BA. The ICA-CBF ratio was defined as ipsilateral ICA flow rate divided by CBF.

### Analyses and statistics

Both ICAs were used in the analysis. We assessed PC-MRI findings for diagnostic analyses with emphasis on conventional stenosis versus near-occlusion, using preoperative CTA as the reference. Post-operative CTA was used as a supplementary reference (see data supplement). As benchmarking for the PC-MRI findings, we assessed the accuracy of individual raters’ CTA diagnoses, compared with consensus CTA diagnosis.

Sample size was derived for including 30 ICAs with findings near the coming threshold, such as severe conventional stenoses, anatomical variations, unclear diagnosis and near-occlusions with modest collapse. This was estimated at occurring at 12.5% of ICAs. We added 15% for likely exclusions or missing post-operative CTAs, resulting in 138 estimated participants.

When applicable, we used mean, standard deviations, median, interquartile ranges, and 95% confidence intervals (CI), *t*-test, Mann-Whitney, one-way ANOVA, Kruskal-Wallis test, kappa values, and intraclass correlation (ICC, single measurement, absolute-agreement, two-way random effect). Diagnostic performance was based on receiver operating characteristics analysis, area under the curve (AUC), and threshold analyses at maximal Youden index and at ⩾95%, ⩾98%, and 100% specificity. Significance level was set at *p* < 0.05. Statistics were performed in SPSS 28.0 (IBM).

## Results

**Figure 2.**
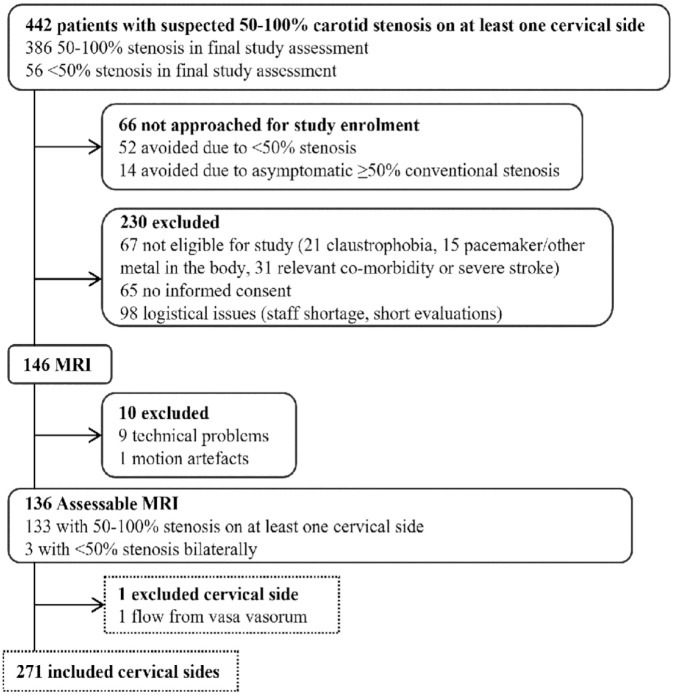
Study flow chart.

We included 136 participants (Figure 2). Baseline characteristics are presented in Table 1. One ICA was excluded due to minute flow in distal ICA from vasa vasorum, which was visible in both CTA and PC-MRI (neither occlusion nor near-occlusion). Therefore, 271 ICAs were available for analysis. The main analysis was conducted on 190 conventional stenoses (102 < 50%, 88 conventional ⩾ 50%) and 49 near-occlusions. Of ⩾50% conventional stenoses, 50 were 50%–69%, 31 were ⩾70% and 7 were too calcified to subtype. In additional analyses, the 20 ICAs with an unclear diagnosis and 12 with occlusion are also assessed. See data supplement for additional baseline aspects.

**Table 1.** Baseline characteristics of participants.

Characteristics	
*N*	136
Age, *mean* (SD)	73 (7)
Women, *n* (%)	43 (32)
Myocardial infarction, *n* (%)	17 (13)
Current angina, *n* (%)	4 (3)
Previous arterial revascularization, *n* (%)	35 (26)
Atrial fibrillation, *n* (%)	17 (13)
Diabetes, *n* (%)	30 (22)
Current smoker, *n* (%)	22 (16)
Systolic blood pressure, *mean mmHg* (SD)	149 (23)
Diastolic blood pressure, *mean mmHg* (SD)	76 (11)
Hypertension^a^, *n* (%)	116 (85)
Symptomatic stenosis: *n* (%)	110 (81)
Presenting event: Stroke *n* (%)	50 (46)
Presenting event: TIA *n* (%)	35 (32)
Presenting event: Retinal *n* (%)	25 (23)
Delay CTA and MRI, *median days* (IQR)	4 (2–7)
Delay surgery and postop CTA, *median days* (IQR)	163 (98–259)

SD: standard deviation; IQR: inter-quartile range.

a Blood pressure >140 systolic, >90 diastolic and/or use of blood pressure reducing medication.

Flow rates and ratios in near-occlusions were lower in ICA and M1, reversed in A1 and Pcom, and higher in BA, P1, and P2 compared with conventional stenoses (Table 2).

**Table 2.** Arterial flow rates and lateral flow rate ratios in stenosis groups.

	<50% stenosis (*n* = 102)	Conventional ⩾50% stenosis (*n* = 88)	Near-occlusion (*n* = 49)	Occlusion (*n* = 12)	*p* ^a^	*p* ^b^	*p* ^c^	AUC^d^ (95% CI)
Mean ipsilateral flow rates, ml/min (SD)								
ICA	251 (68)	206 (71)	65 (47)	0 (0)	<0.001	<0.001	<0.001	0.98 (0.97–1.0)
M1	133 (37)	121 (27)	103 (40)	102 (36)	0.002	<0.001	0.913	0.70 (0.61–0.78)
A1	110 (57)	83 (60)	−9 (42)	−63 (47)	<0.001	<0.001	<0.001	0.94 (0.91–0.97)
A2^e^	59 (18)	53 (18)	55 (24)	63 (22)	0.714	0.165	0.292	0.52 (0.42–0.62)
BA	136 (51)	128 (49)	196 (75)	170 (107)	<0.001	<0.001	0.321	0.77 (0.70–0.84)
P1	44 (29)	53 (32)	107 (46)	88 (73)	<0.001	<0.001	0.281	0.87 (0.81–0.93)
P2	58 (17)	54 (21)	77 (33)	63 (41)	<0.001	<0.001	0.227	0.70 (0.61–0.79)
Pcom	19 (27)	6 (21)	−29 (33)	−36 (41)	<0.001	<0.001	0.552	0.85 (0.78–0.91)
Median flow rate ratios – Ipsilateral compared to contralateral or CBF (IQR)								
ICA-CBF^f^	0.479 (0.415–0.565)	0.369 (0.325–0.431)	0.127 (0.052–0.175)	0 (0–0)	<0.001	<0.001	<0.001	0.99 (0.98–1.0)
ICA	1.48 (1.04–2.93)^g^	0.99 (0.80–1.18)	0.22 (0.10–0.38)	0 (0–0)	<0.001	<0.001	<0.001	0.97 (0.94–1.0)
M1	1.14 (1.03–1.35)	0.97 (0.88–1.13)	0.81 (0.71–0.95)	0.89 (0.62–0.93)	<0.001	<0.001	0.752	0.80 (0.71–0.88)
A1	1.75 (1.00–3.70)	1.00 (0.55–1.61)	0.00 (−0.28–0.08)	−0.33 (−0.45–0.15)	<0.001	<0.001	0.001	0.92 (0.87–0.97)
P1	0.88 (0.41–1.03)	0.97 (0.82–1.03)	1.15 (1.00–1.38)	1.18 (1.00–1.46)	<0.001	<0.001	0.767	0.76 (0.69–0.84)
P2	0.99 (0.84–1.14)	1.00 (0.87–1.05)	1.05 (0.96–1.21)	1.00 (0.82–1.35)	0.002	0.013	0.456	0.64 (0.55–0.72)

AUC: area under the curve; BA: basilar artery; CBF: cerebral blood flow; ICA: internal carotid artery; SD: standard deviation; IQR: inter-quartile range.

Negative number indicate reversed flow.

For flow rates, *T*-test and One-Way ANOVA were used for two- and three-group comparisons. For flow rate ratios, Mann-Whitney and Kruskal-Wallis were used for two- and three-group comparisons.

a Comparing conventional ⩾50% stenoses and near-occlusions.

b Comparing <50% stenosis, conventional ⩾50% stenoses and near-occlusions (three-group comparison).

c Comparing occlusions and near-occlusions.

d Separating near-occlusion and all conventional stenosis (*n* = 190).

e 15 missing MRI measurements due to the A2s being too close together to assess individually.

f ICA-CBF: Ratio between ICA and CBF, where CBF = ICA-ipsi + ICA-contra + BA.

g If excluding cases with contralateral near-occlusion or occlusion: 1.20 (0.90–1.45).

### Near-occlusion and conventional stenosis

**Figure 3.**
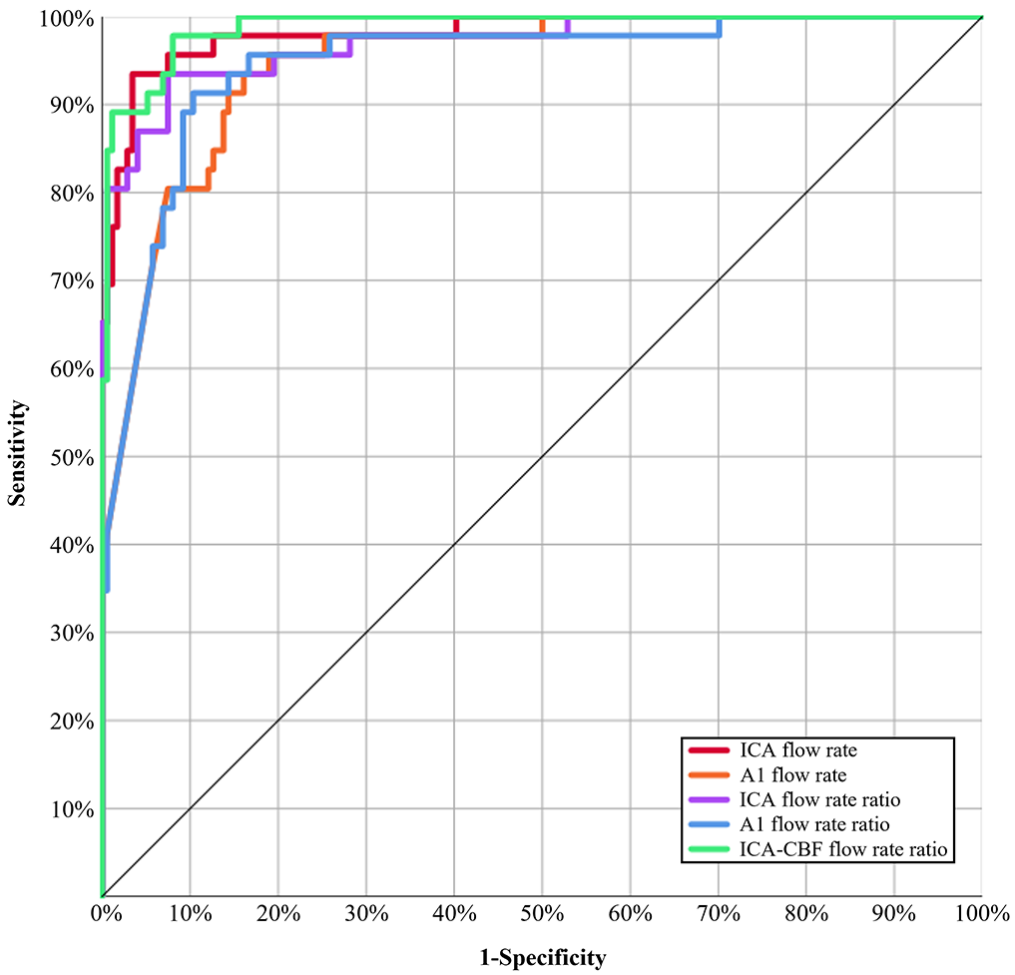
Receiver operating characteristic (ROC) curves. Five best parameters to separate near-occlusion and conventional stenosis.

The highest AUCs were found in ICA flow rate and ICA-CBF ratio (Table 2, Figure 3.). ICA flow rate, ICA-CBF ratio, and both combined, provided similar diagnostic outcomes (Table 3). With best numerical outcomes at ⩾98% specificity, the ICA-CBF ratio ⩽0.225 threshold is henceforth designated as *the best threshold.* When comparing conventional ⩾50% stenosis and near-occlusions (excluding <50% stenosis), the results for the best threshold was virtually unchanged: 90% (44/49) sensitive and 98% specific (86/88). The best threshold correctly identified all <50% stenosis. As both false-positive conventional stenoses ⩾70% stenosis, when comparing conventional ⩾70% stenosis and near-occlusions (excluding <70% stenosis), the best threshold had slightly lower specificity: 94% (29/31). No other parameter had better outcome in this sample. However, both of these false-positive findings had relevant explanations (data supplement). We also assessed the previously suggested thresholds^12^: ⩽110 ml/min ICA flow rate was 84% (41/49) sensitive and 98% specific (187/190). ICA flow rate ratio <0.35 was 74% (34/46) sensitive and 99% specific (173/174).

**Table 3.** Diagnostic performance of thresholds for separating near-occlusion and conventional carotid stenosis based on PC-MRI flow rates.

Goal	Threshold	Sensitivity	Specificity	PPV	NPV
ICA flow rate, ml/min					
*Y* (%)	⩽121	94 (46/49)	96 (183/190)	87 (46/53)	98 (183/186)
⩾95	⩽124	94 (46/49)	95 (181/190)	84 (46/55)	98 (181/184)
⩾98	⩽110	84 (41/49)	98 (186/190)	91 (41/45)	96 (186/194)
100	⩽95	65 (32/49)	100 (190/190)	100 (32/32)	92 (190/207)
ICA-CBF ratio					
*Y* (%)	⩽0.281	98 (48/49)	93 (176/190)	77 (48/62)	99 (176/177)
⩾95	⩽0.254	92 (45/49)	95 (181/190)	83 (44/54)	98 (181/185)
⩾98	⩽0.225^a^	90 (44/49)	99 (188/190)	96 (44/46)	97 (188/193)
100	⩽0.144	57 (28/49)	100 (190/190)	100 (28/28)	90 (190/211)
ICA flow rate *AND/OR* ICA-CBF ratio					
*Y* (%)	⩽120 and/or ⩽0.225	96 (47/49)	97 (184/190)	89 (47/53)	99 (184/186)
⩾95	⩽120 and/or ⩽0.235	96 (47/49)	95 (181/190)	84 (47/56)	99 (181/183)
⩾98	⩽105 and/or ⩽0.225	90 (44/49)	98 (187/190)	94 (44/47)	97 (187/192)
100	⩽95 and/or ⩽0.140	65 (32/49)	100 (190/190)	100 (32/32)	92 (190/207)

CBF: cerebral blood flow; ICA: internal carotid artery; NPV: negative predictive value; PPV: positive predictive value; *Y*: maximum Youden index.

Preoperative CTA used as reference.

a Designated the best threshold with positive likelihood ratio of 85 and negative likelihood ratio of 0.10.

### Separating other degrees of stenosis

With PC-MRI, separation of <50% and conventional ⩾50% stenosis was poor, near-occlusion with and without full collapse had moderate separation but 16% of near-occlusions (all had full collapse) were not distinguishable from occlusion. See online supplement for details, including carotid ultrasound findings.

### Postoperative evaluations

Postoperative CTA was available in 48 ICAs, see data supplemental for detailed description. In short, 75% (9/12) of ICAs with unclear diagnosis were correctly classified with PC-MRI using the best threshold. PC-MRI-findings were similar to preoperative CTA both when preoperative agreed (26/29) and disagreed (5/7) with postoperative CTA.

### Inter-rater agreement of flow rates

The interrater agreements of flow rates were high for both ICA (ICC 0.994, 95% CI 0.991–0.996, *n* = 271) and BA (ICC 0.998, 95% CI 0.997–0.998, *n* = 136). Interrater kappa for the best threshold was 0.98 (95% CI 0.95–1.00).

### Individual CTA observers compared to CTA consensus diagnosis

Interrater interpretations of diagnosing near-occlusion on CTA are found in Table S1. Interrater kappa was 0.70 (95% CI 0.58–0.81) between observer 1 and 2, and 0.58 (95% CI 0.41–0.74) between observer 1 and 3. The sensitivity and specificity for near-occlusion diagnosis compared to final consensus diagnosis were 92% (45/49) and 96% (189/196) for observer 1, 93% (28/30) and 96% (107/112) for observer 2, and 58% (11/19) and 100% (84/84) for observer 3, when differentiating near-occlusion from all other degrees of stenosis.

## Discussion

This study was performed as there is currently no accurate method to diagnose near-occlusion that is feasible for use in routine practice (i.e. threshold-based). The main finding of this study was the excellent diagnostic performance (90% sensitivity and 99% specificity) and interrater reliability (kappa 0.98) of ICA-CBF ratio ⩽0.225 with PC-MRI to separate conventional carotid stenosis and near-occlusion.

### Diagnostic aspects

Except for the small proof-of-concept study of PC-MRI,^12^ PC-MRI (with the best threshold) has better diagnostic performance than any previous near-occlusion threshold of any previous modality^1,2,9–11,17^ and similar performance as individual expert CTA raters compared to consensus CTA diagnosis. The clear advantage with this threshold-approach on PC-MRI compared to the interpretive approach on CTA by experts (our reference test) is feasibility for use in routine practice. The diagnostic performance was also similar or better for near-occlusion with preoperative CTA as reference as any non-invasive modality is for conventional ⩾50% or conventional ⩾70% with conventional angiography as reference.^18^ However, it seems that PC-MRI only separate conventional stenoses and near-occlusions well: PC-MRI failed to separate <50% stenosis and conventional ⩾50% stenosis, a very relevant distinction.^2^ Also, many near-occlusion with full collapse had no detectable flow on PC-MRI. An additional scan sequence with lower velocity encoding (we used 110 cm/s, suitable for artery flow) might resolve this. An alternative for routine practice is to keep this single Venc to shorten camera time and accept that PC-MRI is not the ideal method for separating near-occlusion and occlusion.

No <50% stenosis was mistaken for near-occlusion and when limiting the analysis to those with ⩾50% stenosis, the findings was virtually unchanged. We can therefore conclude that near-occlusion is separated from conventional ⩾50% stenosis with the best threshold. We chose to use conventional stenosis (conventional ⩾50% and <50%) as control group, not conventional ⩾50%, because both should reasonably be separated from near-occlusion for the method to be acceptable; even though conservative treatment is recommended for both near-occlusion and <50% stenosis.^2^ Here, it should be highlighted that four cases with <50% stenosis with A1 aplasia were false-positive with the Youden index threshold for ICA-CBF ratio, why it was relevant to include a control group with <50% stenosis. Both false-positive findings had conventional ⩾70% stenosis, but it rather seemed like other causes than degree of stenosis was the reason for being false positive (mistaken preoperative diagnosis and tandem stenosis).

See also data supplement for minor diagnostic aspects.

### Reliability

The interrater reliability for diagnosing near-occlusion with PC-MRI (kappa 0.98) outperformed that of the CTA raters (0.58–0.70) and for various carotid stenosis approaches in the literature (0.64–0.84, see data supplement). The semi-automatic software likely contributed to this; the observer merely chooses an artery segment for analysis. High reliability despite virtually no joint training and varying neuroradiological expertise between observers underscores the software’s role. Studies assessing PC-MRI test-retest reliability and reliability between PC-MRI software for near-occlusion are warranted.

### Postoperative CTA

In addition to the standard reference, we also used postoperative CTA as recently suggested.^4^ This is not an established method and was only available in selected participants. Rather than a new standard reference, this method should be viewed as providing supplementary information, why most of the information about this method is presented in the data supplement.

ICAs with unclear preoperative diagnosis, only recently first described^7^ were possible to categorize with postoperative CTA. With PC-MRI, these could now be assessed preoperatively for the first time and had an acceptable (75%) accuracy. In previous diagnostic near-occlusion studies, these unclear diagnosis ICAs have been excluded,^12^ merged with the conventional stenosis group^8,10,11^ or not reported separately.^9,17^ Also, there were ICAs where the preoperative assessment did not identify the existence of a distal collapse or mistook the cause of distal collapse, identified by mismatch between preoperative and postoperative CTA diagnoses. We hypothesized that PC-MRI would predict postoperative findings in ICAs with such mismatch and thus be better than preoperative CTA. If true, this could have affected the placement and accuracy of diagnostic thresholds. However, it turned out that PC-MRI rather predicted the preoperative findings in ICAs with mismatch.

### Clinical implications

Conventional ⩾50% stenosis should usually undergo revascularization, near-occlusions should not,^2^ why separating these is relevant in routine practice. Many of our presented thresholds had good outcomes for this separation, with only small numerical differences between them. We chose to emphasize the threshold with the numerically highest specificity that retained high sensitivity (the best threshold) because of the clinical impact: Better if a few near-occlusions are mistaken for conventional stenosis and undergo unnecessary surgery (a small harm by non-perfect sensitivity) than if a few conventional stenoses are mistaken for near-occlusion and surgery is withheld (a larger harm by non-perfect specificity). We foresee a clear role for PC-MRI to separate conventional stenosis and near-occlusion in routine practice and future near-occlusion studies. Currently, near-occlusion is seemingly systematically underdiagnosed,^8,10,11^ why near-occlusion is often perceived to be rare when it is common.^2,19^ Thus, introducing PC-MRI would be a marked improvement for near-occlusion detection to avoid unnecessary surgery. The introduction of PC-MRI will be the first routine indication for PC-MRI in stroke medicine and will likely, over time, lead to a redefinition of near-occlusion from an anatomical to a physiological diagnosis. When introduced, quality control in terms of monitoring the long-term prognosis when patients with symptomatic near-occlusion are conservatively treated is warranted.^4^ Diagnostic validation in additional cohort, especially against a larger sample of >70% stenoses, is warranted. Also, given the issues with PC-MRI, it should be performed in addition to (not instead of) other modalities such as contrast-enhanced MRA, ultrasound or CTA.

### Strengths and limitations

Strengths of this study were a dedicated prospective data collection with pre-specified hypothesis, large sample compared to other PC-MRI studies of carotid stenosis,^12^ expert CTA-assessment as reference test, blinded assessments and first use of postoperative CTA as a supplementary reference method. Limitations include that inclusion was not fully consecutive due to logistical issues. Also, we only included participants with extracranial atherosclerotic stenosis. It is reasonable that patients with other causes of ICA flow reduction, such as severe carotid dissections or carotid T-occlusions, will be indistinguishable from atherosclerotic near-occlusions with our threshold approach to PC-MRI. This again underscores the need to combine PC-MRI with an established modality. We also included a limited number of conventional ⩾70% stenosis. A partial explanation was that many stenoses categorized as uncertain diagnosis would have been conventional ⩾70% stenosis if our two-sided conservative approach had not been used. However, this was both the pre-specified approach and it seemed to be a reasonable choice one given the outcomes among uncertain diagnoses (supplement). While it seems like the main results are applicable to conventional ⩾70% stenosis as well as lesser degrees of stenosis, a larger sample would have been preferable for a firm conclusion about conventional ⩾70% stenosis.

## Conclusion

Internal carotid artery to cerebral blood flow ratio ⩽0.225 on phase contrast MRI is an accurate and reliable method to separate conventional ⩾50% stenosis and near-occlusion that is feasible for routine use. In addition to diagnostic validation (reproduction), studies validating prognostic outcomes are also warranted. Phase contrast MRI should be used in addition to an established modality for grading stenosis as it cannot separate other degrees of stenosis.

## Supplemental Material

sj-docx-1-eso-10.1177_23969873231215634 – Supplemental material for Diagnostic separation of conventional ≽50% carotid stenosis and near-occlusion with phase-contrast MRI Supplemental material, sj-docx-1-eso-10.1177_23969873231215634 for Diagnostic separation of conventional ≽50% carotid stenosis and near-occlusion with phase-contrast MRI by Madelene Holmgren, Alexander Henze, Anders Wåhlin, Anders Eklund, Allan J Fox and Elias Johansson in European Stroke Journal
