# Supplementary material for: Diagnostic separation of conventional ⩾50% carotid stenosis and near-occlusion with phase-contrast MRI
Source: Eur Stroke J. 2023 Nov 30;9(1):135–43. doi: 10.1177/23969873231215634 (PMC10916822; doi:10.1177/23969873231215634)
Supplement: sj-docx-1-eso-10.1177_23969873231215634 – Supplemental material for Diagnostic separation of conventional ≽50% carotid stenosis and near-occlusion with phase-contrast MRI [file sj-docx-1-eso-10.1177_23969873231215634.docx]

**Diagnostic separation of conventional ≥50% carotid stenosis and near-occlusion with phase-contrast MRI**

**SUPPLEMENTAL MATERIALS**

**Background**

***Postoperative CTA***

There are many causes for small distal ICA, and even experts cannot determine the diagnosis when features are too divergent [1]. Approximately 5% of persons with ≥50% stenoses have an unclear cause of small distal ICA [1]. Such ICAs are apparently diagnosable with a recently suggested postoperative CTA approach, where near-occlusion is diagnosed based on the distal ICA increases in size after stenosis removal [2]. Also, expert assessments of preoperative CTA are occasionally incorrect compared to postoperative CTA [2]. Thus, adding postoperative CTA as a supplementary reference method for PC-MRI findings seems reasonable, especially for ICAs with unclear diagnosis of small distal ICA, and ICAs where preoperative and postoperative assessments does not match. In the previous PC-MRI study of near-occlusion, ICAs with unclear diagnosis were excluded, but the need to assess such ICAs was highlighted [3].

**Materials and Methods**

***Preoperative CTA.***

Preoperative CTA was usually done as a part of the clinical routine. As part of the study, CTA was repeated in participants with poor quality of the initial CTA or missing clinical CTA. Various protocols and machines were used at the referring hospital but usually covered aorta to vertex and aimed at the arterial phase. Near-occlusion assessment was done by feature interpretation, using the same approach as in several previous studies [1, 3-4], but here described in more detail:

The whole exam is assessed in order to answer two questions. If both are answered with “yes”, you have a near-occlusion.

1. Is the artery beyond the stenosis reduced in size? That is, is the diameter well beyond the stenosis smaller than expected (is there a collapse)? Here, the border between “yes” and “no” is what is visible, i.e., we did this assessment visually. For reference, a visible difference occurs when ICA ratio (side-to-side ratio) is ≤0.88 [1], and ICA ratio ≤0.87 is 97% sensitive and 89% specific for near-occlusion [5]. When making this assessment, we did not include the naturally larger bulb area (we assessed well beyond the stenosis). In cases with varying distal ICA diameter, we assessed the most representative part, not calling ICA segments that are small only for short distance as being small, such as cases with loops, kinks, fibromuscular dysplasia and/or (seemingly chronic) dissections.
2. Is a proximal severe stenosis the most reasonable cause for the small distal ICA? Thus, is the stenosis sufficiently severe and are there other causes? Other causes aspect refers foremost to anatomical variation of distal ICA size, where (unlike loops and kinks) the entire extracranial distal ICA is smaller than contralateral ICA. This associated with asymmetry in the Circle of Willis. This exists in 8% of persons without steno-occlusive disease in all age-groups and is foremost caused by a small or not visible A1 segment on the side of the smaller ICA [1]. However, a small A1 segment can also be caused by collateral use in a near-occlusion. Therefore, assessment of Circle of Willis is limited to if there is reason to suspect anatomical variation. Other causes of small distal ICA that were always considered were tandem lesions and ICA hypoplasia (but we had no such cases in this sample). However, it is unclear how severe a stenosis needs to be to cause distal ICA collapse. Diameter ≤1.3 mm is 90% sensitive and 84% specific [5], and 49% of near-occlusions have such tiny residual lumen that contrast opacity is reduced on CTA, presumably due to partial volume effect [6]. Rather, in absence of an alternative explanation a lesser stenosis severity can be accepted and vice versa. Beyond stenosis severity, the extent of distal collapse is often more severe in near-occlusions than in anatomical variations. Here, ICA ratio, absolute distal ICA diameter and ICA to ECA comparison (ECA ratio) are useful. In persons without steno-occlusive disease (n=257), mean distal ICA diameter is 4.1 mm (SD 0.5) [1], why 95% of persons have ≥3.3 mm distal ICA diameter (mean + 1.645 SD, one-tailed approach). Distal ICA diameter ≤3.5 mm is 95% sensitive and 90% specific for near-occlusion [5]. ECA should be assessed just before its terminal bifurcation (usually just behind the jaw at C2 vertebrae level). Foremost near-occlusions causes sufficient distal ICA collapse for ECA ratio to approach (or be smaller than) 1. ECA ratio ≤1.27 is 91% sensitive and 87% specific for near-occlusion [5].

The four main metrics do describe near-occlusion is stenosis severity, ICA ratio, ICA diameter and ECA ratio [5]. Each metric has its own pitfalls: Stenosis severity is problematic given the risk of circular logic. Also, a variation between cases can perhaps be explained by that the distal ICA collapse is strongly associated with flow [3]. Reasonably, the better the collateral capacity, the easier it will be for blood flow to use to collateral pathways, and the lesser the stenosis severity needs to be to cause flow reduction and reduced distal ICA diameter. Stenosis severity is also missing in cases with severe calcifications. ICA ratio is false positive for other causes of small distal ICA, false negative when the contralateral ICA is also small due to bilateral near-occlusion or anatomical variance, and ICA ratio is missing in contralateral occlusion. ICA diameter can be false positive for other causes, and false negative in persons with large native ICAs. ECA ratio can be false positive in cases with native large ECAs and false negative in cases with CCA and bulb stenosis – causing ECA collapse as well, which can be confirmed by side-to-side comparison of ECA diameters.

Given all pitfalls, the diagnosis was made by synthesis of all parts of the exam. This is an expert-approach to separate near-occlusion from conventional stenoses. While reasonably state-of-the-art, it is reasonably too complicated for wide-spread routine use – why the need for easily applied threshold techniques. It should be noted that we did not use these cited thresholds as criteria, but rather as guides: Each feature was assessed on a spectrum, not as yes/no. Missing information could be accepted, but other features must then be more convincing for the diagnosis to be sufficiently clear. We had several cases where no diagnosis was sufficiently clear – these were designated as unclear diagnosis. As presented, no metric is both very sensitive and specific. Furthermore, the cited diagnostic outcomes are from a study when the assessor did both feature interpretation and measurements [5], which is reasonable if one attempts feature interpretation and needs a guide. However, blind application of these metrics is not recommended. A recent study compared with CTA measurements blinded to criteria-based diagnosis on conventional angiography as reference. Then, none of these four metrics was >75% sensitive and specific for 2 of 2 observers [7].

In arterial segments with tiny lumen and lower (darker) radiodensity than surrounding arteries (presumably due to partial volume effect), we assigned a .5 mm diameter when the contrast was visible and .2 mm when not visible (but clearly existent from context).

***Postoperative CTA***

Postoperative CTA was occasionally performed due to postoperative symptoms, but foremost as part of the study in participants that underwent carotid surgery or stenting. Study postoperative CTA was aimed at three months after surgery or stenting but was occasionally prolonged due to Covid19 considerations (elderly persons not examined for research, especially before vaccinations). For ethical concerns regarding radiation exposure, postoperative CTA was limited to participants with possibly asymmetric ICAs of any cause, i.e., participants treating an ICA with near-occlusions, unclear diagnosis or conventional stenosis with asymmetric ICAs due to anatomical variance.

All postoperative CTAs were assessed by EJ, and a subset by AJF, who were blinded to each other, MRI-finding, and their preoperative assessment. To ensure blinding, postoperative assessments were done several months after preoperative assessments were registered. Disagreements were resolved by consensus discussion. Exams with poor quality, severe residual stenosis, and/or a stent covering the whole distal ICA were excluded. The postoperative CTA was displayed side-by-side with the preoperative CTA to ensure the ability to assess change in distal ICA diameter. Near-occlusion was defined as an apparent visible increase in ipsilateral distal ICA diameter on the postoperative CTA compared to the preoperative CTA.

***MRI protocol & post-processing workflow***

The MRI scans were performed on a 3T scanner (GE Discovery MR 750, Milwaukee, WI, USA) with a 32-channel head coil. The four-dimensional flow MRI sequence provided time-resolved velocities in three directions, 20 frames per cardiac cycle [8-9]. No contrast agent was used in the MRI scans. Input imaging parameters were: five-point balance velocity encoding (venc) 110 cm/s, TR/TE 6.5/2.7 ms, flip angle 8°, 16000 radial projections, acquisition resolution 300×300×300, reconstructed resolution 320×320×320, imaging volume 220×220×220 mm^3^ isotropic voxel size .69 mm. The scan time sequence was ≈9 minutes and provided a full brain coverage of velocities in three spatial directions.

Blood flow rates were quantified with on MATLAB (R2020b, The MathWorks Inc., Natick, MA, USA). Vessel identification and area segmentation were based on angiographic complex difference images. The arterial segment of interest was manually selected from the angiogram. Arterial lumen was segmented from perpendicular cross-sectional planes, using a maximum image intensity threshold locally in each plane [10]. The flow rate in each artery was calculated as the average velocity multiplied by the cross-sectional area in each plane and averaging 15 consecutive planes. The threshold parameter of initially 16% was iteratively increased in steps of 1%, up to maximum 24%, if the coefficient of variation between the flow rates from the cut-planes was >15% (a step added for this study). This was rarely necessary for the larger arteries (ICA, BA, M1). All final cut-planes were inspected, and planes with a nearby artery still persistent in the image were excluded.

Some A2s were too close to one another and, therefore, not separable. In such instances, A2s were assessed with a previously published approach [11], using the same angiographic images, but applying an image intensity threshold based on the whole image volume, without using perpendicular planes. Some arterial segments were visible in the MR angiograms but not interpretable in the post-processing. Based on the angiogram signal, these were set to approximated flow rates of 10 ml/min (weak signal) or 20 ml/min (strong signal). Arterial segments not visible in the angiogram were set to 0 ml/min.

**Results**

***Baseline***

Of the 190 conventional stenoses, 67 (35%) had <0% stenosis, 13 (7%) had 1-30% stenosis, 22 (12%) had 31-49% stenosis, 51 (27%) had 50-69% stenosis, 31 (16%) had ≥70% stenosis and 7 (4%) had a too calcified stenosis to be measured, but reasonably >50%. Here, <0% stenosis means that either no plaque or minor plaques in the anatomically larger carotid bulb that resulted in the lumen being wider than the distal ICA.

There were 6 preoperative recurrent strokes among the 110 participants with symptomatic ≥50% stenosis, of which 1 had near-occlusion.

***<50% stenosis and conventional ≥50% stenosis***

For separating <50% stenoses from conventional ≥50% stenoses, ICA flow rates had AUC .70 (95% CI .62-.77) and ICA-CBF ratio had AUC .74 (95% CI .66-.81). Threshold for highest Youden index was ≤.403 for ICA-CBF ratio, resulting in 67% (59/88) sensitivity and 79% (81/102) specificity for conventional ≥50% stenosis. Exclusion of those with contralateral near-occlusion or occlusion had no relevant impact on these findings.

***Near-occlusion with and without full collapse***

In separating near-occlusion with and without full collapse, ICA flow rate (AUC .95, 95% CI .88-1.0) and ICA-CBF ratio (AUC .94, 95% CI .88-1.0) provided high diagnostic performance. ICA flow rates had numerically slightly better performance than ICA-CBF ratio and combinations of these two parameters. Threshold for highest Youden index was ICA flow rate ≤38 ml/min, which was 85% (11/13) sensitive and 92% (33/36) specific for near-occlusion with full-collapse.

***Near-occlusion and occlusion***

None of the occlusions had flow in their external ICA on PC-MRI. There were 8 ICAs without occlusion that also had no visible ICA flow on PC-MRI, all were near-occlusion with full collapse. Thus, 62% of near-occlusions with full collapse and 16% of near-occlusions were not separable from occlusion.

***Postoperative CTA***

Postoperative CTA was available in 48 ICAs from 47 participants (1 underwent bilateral surgery).

There were 20 ICAs with uncertain diagnosis on preoperative CTA, 12 had post-operative CTA (25% of post-operative CTAs). Of these, 7 were conventional stenoses on postoperative CTA, with PC-MRI findings of median (range) 161 (91-199) ml/min ICA flow rate and .343 (.195-.537) ICA-CBF ratio. Best threshold accurately classified 6/7 (86% specificity). Remaining 5 were near-occlusion on postoperative CT, with PC-MRI findings of median (range) 94 (63-145) ml/min ICA flow rate and .216 (.140-.286) ICA-CBF ratio. Best threshold accurately classified 3/5 (60% sensitivity). No other threshold increased accuracy compared to best threshold.

If the traditional 1-sided conservative approach to CTA grading was used, i.e. considering all uncertain diagnoses as conventional stenosis [4, 13-15], 70% (14/20) of uncertain ICAs would have been conventional ≥70%, 3 (15%) 50-69% and 3 (15%) too calcified to assess. Of these 20, best threshold on PC-MRI was positive in 8, but only one (13%) could be confirmed as truly false positive on post-operative CTA (an 87% stenosis). 4 lacked post-operative CTA (3 conventional ≥70% stenosis, 1 too calcified) and 3 were actually not false positives, i.e. had near-occlusion on post-operative CTA (1 50-69%, 1 ≥70% and 1 too calcified). Of 12 negatives, 6 (50%) could be confirmed on post-operative CTA (1 50-69%, 4 conventional ≥70% and 1 too calcified). 4 lacked post-operative CTA (1 50-69%, 2 conventional ≥70% and 1 too calcified) and 2 were actually not true negatives, i.e. had near-occlusion on post-operative CTA (1 conventional ≥70% and 1 too calcified). Hence, a 1-sided approach would lead to a 45% increase in the sample of conventional ≥70% stenosis (from 31 to 45), a drop in specificity from 99% (188/190) to 95% (200/210) but only 1 of the 8 additional “false positives” could be confirmed on post-operative CTA.

In 29 ICAs (60%), pre- and postoperative CTA diagnosis agreed, with PC-MRI being correct in 7/8 conventional stenoses and 19/21 near-occlusions. In 7 ICAs (15%), pre- and postoperative CTA diagnosis disagreed. Of these, 3 were conventional stenoses according to postoperative CTA, with PC-MRI findings of median (range) 149 (76-189) ml/min ICA flow rate and .223 (.140-.323) ICA-CBF ratio. Best threshold accurately classified 1/3 (33% specificity). Of these three, two were seemingly caused by anatomical variation (small ipsilateral A1), one had varying distal ICA diameter that would have been near-occlusion if the smaller aspect was considered as representative – but it did not change after stenosis removal. Remaining 4 were near-occlusion according to postoperative CTA, with PC-MRI findings of median (range) 145 (96-190) ml/min ICA flow rate and .300 (.149-.359) ICA-CBF ratio. One of these four was correctly identified by PC-MRI, i.e. best threshold had 25% sensitivity. This case was an 81% stenosis categorized as uncertain by one observer but anatomical variant by the other and consensus was variant. This was one of two false-positives in main assessment of best threshold. One had distal ICA diameter 3.8 mm and an even more severe near-occlusion on contralateral side (distal ICA diameter 2.7 mm), two were considered anatomical variants (one small A1, one contralateral fetal PCA), both with quite typical (apparently misleading) markers against near-occlusion: not very severe stenosis (1.1-1.5 mm), distal ICA diameter 3.6-3.9 mm, ICA-ratio 0.83-0.88 and ECA-ratio 1.64-1.77. Beyond re-assignment of a single ICA, no other threshold increased accuracy compared to best threshold.

Postoperative CTA was available in 5/7 disagreements (3/5 near-occlusion, 2/2 conventional stenoses) between preoperative CTA and best threshold of PC-MRI (ICA-CBF ratio ≤.225). Of these, 2/5 (40%) were misclassified on preoperative CTA (postoperative CTA and PC-MRI agreed on 1 near-occlusion, 1 conventional stenosis), and 3/5 (60%) were misclassified on PC-MRI (pre- and postoperative CTA agreed on 2 near-occlusions and 1 conventional stenosis).

***Likely causes for disagreements between preoperative CTA and PC-MRI***

The likely causes of the 7 disagreements between best threshold of PC-MRI and preoperative CTA were identified.

The two false positive near-occlusions both had conventional ≥70% stenosis on preoperative CTA. One of these was an aforementioned 81% stenosis misclassification as anatomical variance on preoperative CTA (was near-occlusion on post-operative CTA), ICA-CBF ratio was .149. The other was confirmed as not near-occlusion on postoperative CTA, an unilateral 73% stenosis with symmetric ICAs both before and after CEA. The ipsilateral ICA was clearly smaller on PC-MRI angiogram with 112ml/min compared to 269 ml/min in contralateral ICA, ICA-CBF ratio .210. Seemingly, this was caused by tandem lesion (a stenosis in cavernous segment). Also, A1 was not visible on PC-MRI but was visible on CTA. However, of remaining 26 A1s not visible on PC-MRI in this study, 12 (46%) were visible on CTA.

There were five false negative near-occlusions. One was an aforementioned misclassification as near-occlusion on pre-operative CTA (was not near-occlusion on post-operative CTA), one CTA observer thought it was a near-occlusion, the other that it was unclear. ICA-CBF ratio was .323. The other four was likely because of overlap between cases, 2 were confirmed near-occlusions on post-operative CTA, 2 lacked post-operative CTA, all 4 had ICA-CBF ratio between .254-.279.

With the Youden index-threshold for ICA-CBF ratio (≤.281), the only missed near-occlusion was the aforementioned conventional stenosis mistaken for near-occlusion on pre-operative CTA. However, the Youden-index threshold resulted in 12 additional conventional stenoses being classified as near-occlusions compared to best threshold: One was near-occlusion on post-operative CTA, remaining 11 lacked post-operative CTA. One of these 11 was possibly a near-occlusion on the merit of being the only of 190 (0.5%) conventional stenoses with reversed A1 flow on PC-MRI, which was seen in 39% of near-occlusions. In remaining 10, the likely cause for false positive ICA-CBF ratio was A1-assymetry: The ipsilateral A1 was small with antegrade low (n=6, median A1 flow rate ratio .26) or aplastic (n=5, not visible in either CTA or PC-MRI). Noteworthy is that 4/5 cases with A1 aplasia had no relevant ipsilateral stenosis, but all 6 with small A1 had ≥50% stenosis.

***Carotid ultrasound***

All but 4 participants underwent carotid ultrasound. Mean peak systolic velocity was 213 (SD 120) cm/s in ICAs with conventional ≥50% stenosis on CTA and 367 (SD 137) among near-occlusions (p<0.001). Mean end-diastolic velocity was 76 (SD 67) cm/s in ICAs with conventional ≥50% stenosis on CTA and 137 (SD 71) among near-occlusions (p<0.001). Categorical comparisons of ultrasound and CTA findings are presented in supplemental table 2. Only 3 (6%) of near-occlusions on CTA were detected by ultrasound as low flow velocity in a severe stenosis, 2 had not detectable flow on PC-MRI, 1 had ICA-CBF ratio of 0.097. Ultrasound also had one false-positive near-occlusion that was occluded on CTA. There were 42 ICAs with positive best threshold on PC-MRI where ultrasound categorized the case as conventional stenosis. Of these, 34 (81%) were near-occlusions and 6 (14%) were uncertain diagnoses on CTA. 2 (5%) were false positive PC-MRI according to both CTA and ultrasound, categorized as conventional ≥70% stenosis on both modalities.

**Discussion**

***Minor diagnostic aspects***

The previously suggested PC-MRI thresholds were 100% accurate when derived but had worse performance in our material [3]. However, the underlying data in both studies seems to be very similar: Based on their online supplement figure 2, our Youden index flow rate ICA threshold (≤121 ml/min, 94% sensitive and 96% specific in our study) would also have been 100% accurate in the previous study [3].

It seems reasonable that even the modest separation between ≥50% conventional stenosis and <50% is an overestimation – the AUC is likely not separable from .5 in a real-life scenario. We had only 3 participants with bilateral <50% stenosis, why those with <50% most often had a contralateral stenosis. Thus, our <50% population is not the intended population for separating <50% and conventional ≥50% stenosis. That our population was skewed towards showing an affect where none existed is reasonable given that ICA flow rate ratio in those with <50% stenosis was >1.0 (as in contralateral lower flow). In persons with bilateral <50% stenosis, 1.0 ICA flow rate ratio would be expected on average. This finding also remained after excluding participants with contralateral near-occlusion and occlusions.

While separation of near-occlusion with and without full collapse does not impact recommended management today [16], recent studies have suggested that some type of treatment might be indicated for near-occlusion with full collapse [12]. PC-MRI was reasonably accurate (85% sensitivity and 92% specificity) for full collapse, but the alternative (CTA) is more feasible than PC-MRI. Too few participants (n=1) with near-occlusion and recurrent stroke to create a prognosis-driven definition of full collapse in a manner like how the CTA-based definition of full collapse was created [12].

As previously presented [13], carotid ultrasound has a very limited role in near-occlusion diagnostics as most near-occlusions have high velocity in the stenosis.

***Carotid stenosis reliability in the literature***

Our interrater kappa with PC-MRI for near-occlusion was .98. This was higher than previous near-occlusion approaches in the literature: .80 for feature interpretation (like current approach) [14], .46-.83 for interpreting single features [7, 16], .78-.84 for combinations of interpreted features [7, 16**]**, and .64-.84 for combination of measurements [7, 17]. Our interrater reliability was also higher than for grouping conventional stenoses into clinically relevant groups with conventional angiography (.72) **[**18], CTA (.75) [19] and contrast-enhanced magnetic resonance angiography (CE-MRA, .84) [20].

**Supplement references**

1. Johansson E, Aviv RI, Fox AJ. Atherosclerotic ICA stenosis coinciding with ICA asymmetry associated with Circle of Willis variations can mimic near-occlusion. Neuroradiology 2020;62:101–104

2. Johansson E, Holmgren M, Henze A et al. Diagnosing carotid near-occlusion is a difficult task—but it might get easier. *Neuroradiology* 2022;64:1709–1714.

3. Johansson E, Zarrinkoob L, Wåhlin A et al. Diagnosing carotid near-occlusion with phase-contrast MRI. *AJNR Am J Neuroradiol* 2021;42:927–929.

4. Johansson E, Gu T, Aviv RI et al. Carotid near-occlusion is often overlooked when CT angiography is assessed in routine practice. Eur Radiol k2020;30:2543–2551.

5. Bartlett ES, Walters TD, Symons SP, Fox AJ. Diagnosing carotid stenosis near-occlusion by using CT angiography. *AJNR Am J Neuroradiol* 2006;27:632-637

6. Kellomäki E, Gu T, Fox AJ, Johansson E. Symptomatic and asymptomatic carotid near-occlusions have very similar angiographic appearance on CT-angiography. *Neuroradiology* 2022;64:2203-2206.

7. Manrique-Zegarra M, García-Pastor A, Castro-Reyes E, et al. CT angiography for diagnosis of carotid near‑occlusion: a digital subtraction angiography validation study. *Neuroradiology* 2022;64:1729–1735.

8. Gu T, Korosec FR, Block WF et al. PC VIPR: A high-speed 3D phase-contrast method for flow quantification and high-resolution angiography. *AJNR Am J Neuroradiol* 2005;26:743–749.

9. Johnson KM, Markl M. Improved SNR in phase contrast velocimetry with five-point balanced flow encoding. *Magn Reson Med* 2010;63:349–355.

10. Holmgren M, Wåhlin A, Dunås T et al. Assessment of cerebral blood flow pulsatility and cerebral arterial compliance with 4D flow MRI. *J Magn Reson Imaging* 2020;51:1516–1525.

11. Wåhlin A, Ambarki K, Birgander R et al. Measuring pulsatile flow in cerebral arteries using 4D phase-contrast MR imaging. *AJNR Am J Neuroradiol* 2013;34:1740–1745

12. Johansson E, Gu T, Fox AJ. Defining carotid near-occlusion with full collapse: a pooled analysis. *Neuroradiology* 2022;64:59–67.

13. Johansson E, Vanoli D, Bråten-Johansson I et al. Near-occlusion is difficult to diagnose with common carotid ultrasound methods. Neuroradiology 2021;63:721–730.

14. Gu T, Aviv RI, Fox AJ, Johansson E. Symptomatic carotid near‑occlusion causes a high risk of recurrent ipsilateral ischemic stroke. *J Neurol* 2020;267:522–530

15. Naylor R, Rantner B, Ancetti S et al. European Society for Vascular Surgery (ESVS) 2023 Clinical Practice Guidelines on the Management of Atherosclerotic Carotid and Vertebral Artery Disease. *Eur J Vasc Endovasc Surg* 2023;65:7–111.

16. Fox AJ, Eliasziw M, Rothwell PM, et al. Identification, Prognosis, and Management of Patients with Carotid Artery Near Occlusion. *AJNR Am J Neuroradiol* 2005;26:2086–2094

17. Johansson E, Öhman K, Wester P. Symptomatic carotid near-occlusion with full collapse might cause a very high risk of stroke. *J Intern Med* 2015;277:615-623

18. Rothwell PM, Gibson RJ, Slattery J, Warlow CP. Prognostic value and reproducibility of measurements of carotid stenosis. A comparison of three methods on 1001 angiograms. European Carotid Surgery Trialists' Collaborative Group. *Stroke* 1994;25:2440-2444

19. Howard P, Bartlett ES, Symons SP, Fox AJ, Aviv RI. Measurement of carotid stenosis on computed tomographic angiography: reliability depends on postprocessing technique. *Can Assoc Radiol J* 2010;61:127-132

20. U-King-Im JM, Trivedi RA, Cross JJ, et al. Measuring carotid stenosis on contrast-enhanced magnetic resonance angiography: diagnostic performance and reproducibility of 3 different methods. *Stroke* 2004;35:2083-2088

**Supplemental Table 1** Interrater agreement of CTA assessments.

|  | | Conventional | Unclear | Near-occlusion | Occlusion | Total |
| --- | --- | --- | --- | --- | --- | --- |
| Observer 1 | | | | | | |
| Observer 2 | Conventional | 86 | 2 | 4 | 0 | **92** |
|  | Unclear | 5 | 1 | 3 | 0 | **9** |
|  | Near-occlusion | 3 | 2 | 25 | 3 | **33** |
|  | Occlusion | 0 | 0 | 0 | 8 | **8** |
|  | **Total** | **94** | **5** | **32** | **11** | **142** |
| Observer 1 | | | | | | |
| Observer 3 | Conventional | 68 | 9 | 2 | 0 | **79** |
|  | Unclear | 0 | 3 | 8 | 0 | **11** |
|  | Near-occlusion | 0 | 1 | 10 | 0 | **11** |
|  | Occlusion | 0 | 0 | 0 | 2 | **2** |
|  | Total | **68** | **13** | **20** | **2** | **103** |
| Final consensus diagnosis | | | | | | |
| Observer 1 | Conventional | 158 | 3 | 1 | 0 | **162** |
|  | Unclear | 5 | 11 | 2 | 0 | **18** |
|  | Near-occlusion | 1 | 6 | 45 | 0 | **52** |
|  | Occlusion | 0 | 0 | 1 | 12 | **13** |
|  | **Total** | **164** | **20** | **49** | **12** | **245** |
| Final consensus diagnosis | | | | | | |
| Observer 2 | Conventional | 88 | 3 | 1 | 0 | **92** |
|  | Unclear | 4 | 4 | 1 | 0 | **9** |
|  | Near-occlusion | 1 | 2 | 28 | 2 | **33** |
|  | Occlusion | 0 | 0 | 0 | 8 | **8** |
|  | **Total** | **93** | **9** | **30** | **10** | **142** |
| Final consensus diagnosis | | | | | | |
| Observer 3 | Conventional | 70 | 8 | 1 | 0 | **79** |
|  | Unclear | 1 | 3 | 7 | 0 | **11** |
|  | Near-occlusion | 0 | 0 | 11 | 0 | **11** |
|  | Occlusion | 0 | 0 | 0 | 2 | **2** |
|  | **Total** | **71** | **11** | **19** | **2** | **103** |

26 cervical sides from 13 participants excluded as these were only assessed by observer 1, all with conventional stenosis and no suspicion of near-occlusion.

|  | | CTA | | | | | | | |
| --- | --- | --- | --- | --- | --- | --- | --- | --- | --- |
|  |  | <50% | 50-69% | Calc ^a^ | ≥70%^b^ | Uncertain | Near-occlusion | Occlusion | All |
| Ultrasound | <50% ^c^ | 0/93 | 0/21 | 0/1 | 0/3 | 0/0 | 0/1 | 0/0 | 0/119 |
|  | 50-69%^c^ | 0/3 | 0/23 | 0/0 | 0/15 | 0/6 | 3/2 | 0/0 | 3/49 |
|  | ≥70% ^c^ | 0/1 | 0/6 | 0/6 | 2/10 | 6/6 | 31/2 | 0/0 | 39/31 |
|  | Near-occlusion^c^ | 0/0 | 0/0 | 0/0 | 0/0 | 0/0 | 3/0 | 1/0 | 4/0 |
|  | Occlusion | 0/0 | 0/0 | 0/0 | 0/0 | 0/0 | 5/0 | 10/0 | 15/0 |
|  | Poor quality | 0/0 | 0/0 | 0/0 | 0/0 | 1/0 | 2/0 | 0/0 | 3/0 |
|  | Not done | 0/5 | 0/0 | 0/0 | 0/1 | 1/0 | 0/0 | 0/0 | 2/6 |
|  | All | 0/102 | 0/50 | 0/7 | 2/29 | 8/12 | 44/5 | 12/0 | 66/205 |
| Numbers represent if best threshold for PC-MRA was positive/negative. Note that this threshold includes 0, why occlusions are positive.  ^a^ Too calcified to separate 50-69% and ≥70% stenosis,  ^b^ Refers to conventional stenoses  ^c^ Based on local ultrasound criteria (previously validated with conventional angiography), where ICA PSV 145-240 cm/s is 50-69% stenosis, >240 cm/s is ≥70% conventional stenosis and near-occlusion is diagnosed when ICA PSV is <145 cm/s and stenosis severity on B-mode is used to distinguish from <50% stenosis. | | | | | | | | | |

**Supplemental Table 2** Comparison of CTA, ultrasound and PC-MRI
